# Cognitively Engaging Activity Is Associated with Greater Cortical and Subcortical Volumes

**DOI:** 10.3389/fnagi.2016.00094

**Published:** 2016-05-02

**Authors:** Talia R. Seider, Robert A. Fieo, Andrew O’Shea, Eric C. Porges, Adam J. Woods, Ronald A. Cohen

**Affiliations:** ^1^Center for Cognitive Aging and Memory, Department of Aging and Geriatric Research, Institute on Aging, University of FloridaGainesville, FL, USA; ^2^Department of Clinical and Health Psychology, University of FloridaGainesville, FL, USA

**Keywords:** cognitive aging, cognitive activity, healthy aging, brain volume, MRI, gray matter, social activity, physical activity

## Abstract

As the population ages and dementia becomes a growing healthcare concern, it is increasingly important to identify targets for intervention to delay or attenuate cognitive decline. Research has shown that the most successful interventions aim at altering lifestyle factors. Thus, this study examined how involvement in physical, cognitive, and social activity is related to brain structure in older adults. Sixty-five adults (mean age = 71.4 years, standard deviation = 8.9) received the Community Healthy Activities Model Program for Seniors (CHAMPS), a questionnaire that polls everyday activities in which older adults may be involved, and also underwent structural magnetic resonance imaging. Stepwise regression with backward selection was used to predict weekly time spent in either social, cognitive, light physical, or heavy physical activity from the volume of one of the cortical or subcortical regions of interest (corrected by intracranial volume) as well as age, education, and gender as control variables. Regressions revealed that more time spent in cognitive activity was associated with greater volumes of all brain regions studied: total cortex (β = 0.289, *p* = 0.014), frontal (β = 0.276, *p* = 0.019), parietal (β = 0.305, *p* = 0.009), temporal (β = 0.275, *p* = 0.020), and occipital (β = 0.256, *p* = 0.030) lobes, and thalamus (β = 0.310, *p* = 0.010), caudate (β = 0.233, *p* = 0.049), hippocampus (β = 0.286, *p* = 0.017), and amygdala (β = 0.336, *p* = 0.004). These effects remained even after accounting for the positive association between cognitive activity and education. No other activity variable was associated with brain volumes. Results indicate that time spent in cognitively engaging activity is associated with greater cortical and subcortical brain volume. Findings suggest that interventions aimed at increasing levels of cognitive activity may delay cognitive consequences of aging and decrease the risk of developing dementia.

## Introduction

Older age is the primary risk factor for neurodegenerative diseases such as Alzheimer’s disease (AD). As the size and proportion of the population over age 65 increases, the number of people with dementia is expected to increase substantially, raising healthcare costs and caregiver burden (Alzheimer’s Association, 2015). Thus, it is becoming increasingly important to identify targets for intervention with the aims of delaying or attenuating cognitive decline.

While several pharmaceuticals exist to delay cognitive decline, research has shown that the best results come from interventions aimed at altering lifestyle factors (Williams et al., 2010; Imtiaz et al., 2014). Greater self-reported levels of engagement in cognitive, social, and physical activity have frequently been associated with higher cognitive functioning scores (Barnes et al., 2004; Newson and Kemps, 2005; McGue and Christensen, 2007; Vemuri et al., 2012; Opdebeeck et al., 2016). Furthermore, physical activity and cognitive engagement are among the only factors consistently associated with decreased risk for AD and cognitive decline (Fratiglioni et al., 2004; Kramer et al., 2004; Hertzog et al., 2009; Williams et al., 2010). Still, the mechanisms of such effects remain poorly understood.

Brain structure and function presumably mediate the link between an active lifestyle and reduced risk for cognitive decline. Observational studies have shown that increased levels of physical activity are associated with larger brain volumes, especially in frontal and hippocampal areas (Rovio et al., 2010; Bugg and Head, 2011; Doi et al., 2015), and interventional studies have shown that physical activity can increase hippocampal (Erickson et al., 2011) and frontal volumes (Colcombe et al., 2006).

Research on the association between cognitive or social engagement and brain volume is less extensive. Proxy measures of cognitive reserve, such as intellectual attainment, have been linked with greater brain volume (Stern, 2009). New learning has been shown to cause increased parietal and hippocampal size in young adults (Draganski et al., 2006), and cognitive training has been associated with increased hippocampal volume and preserved white matter integrity in older adults (Engvig et al., 2014). Greater frequency of cognitive leisure activities has been associated with larger gray matter volume in frontal and limbic regions (Schultz et al., 2015), while higher scores on measures combining cognitive and social activities have been related to more normal-appearing white matter (Gow et al., 2012a) and reduced hippocampal decline over time (Valenzuela et al., 2008). More self-reported social engagement has also been associated with greater temporal and occipital gray matter volume (James et al., 2012), and an intervention study reported that increase in social activity was associated with increased total brain volume (Mortimer et al., 2012). Still, other research has shown no relationship between cognitive or social activity and volumetric data (Foubert-Samier et al., 2012; Vaughan et al., 2014; Van der Vegt, 2015), and the link between these lifestyle factors and regional cerebral volumes remains understudied.

The purpose of this study was to examine how physical, cognitive, and social activity is related to brain structure. Levels of engagement in everyday activities was measured via self-report. Based on previous research, we generally expected higher self-reported levels of physical, social, and cognitive activity to be associated with greater volumes, especially in frontal and limbic regions for physical activity, temporal and occipital regions for social activity, and parietal, frontal, and limbic regions for cognitive activity.

## Materials and Methods

### Participants

Sixty-five community dwelling individuals in the Gainesville and North Florida region were recruited to complete a magnetic resonance imaging (MRI) scan and a cognitive assessment, including the Montreal Cognitive Assessment (MoCA), a brief screen of cognitive functioning. Exclusion criteria included history of head injury with loss of consciousness greater than 20 min, neurologic condition such as dementia, epilepsy, or stroke, major psychiatric illness such as schizophrenia or bipolar disorder, inability to undergo MRI, and MoCA score less than 20. Sample demographics and characteristics are listed in **Table 1**. Participants had a mean age of 71, they were generally well educated with a mean education of 17 years, slightly more than half were females, they were mostly Caucasian, and they were generally cognitively intact with a mean MoCA score of 26. The study was approved by University of Florida Institutional Review Board and written informed consent was obtained from all study participants.

**Table 1:** Sample characteristics (*N* = 65).

	Mean (SD)	Range
Age (years)	71.4 (8.9)	48–85
Education (years)	16.8 (2.5)	12–20
% male	43.1	
% Caucasian	95.4	
MoCA	26.0 (2.7)	20–30

*MoCA, Montreal Cognitive Assessment.*

### Activity Assessment

**FIGURE 1:**
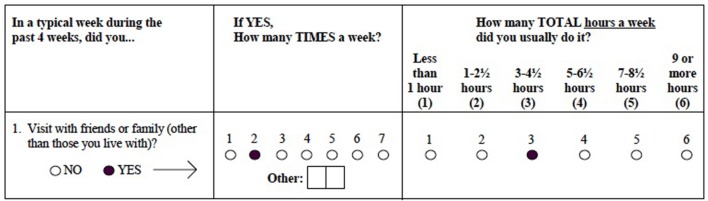
**Sample Community Healthy Activities Model Program for Seniors (CHAMPS) test item**.

The Community Healthy Activities Model Program for Seniors (CHAMPS) questionnaire was developed as part of an intervention study aimed at increasing participation in physical activities in community dwelling elderly. It was designed to measure current levels of energy expenditure by taking a poll of everyday activities in which older adults may be involved (Stewart et al., 1997). Participants were asked whether or not they engaged in a particular activity during a typical week in the past month. If they had, they were asked to fill in the number of times they engaged in the activity per week and mark the total number of hours spent in the activity per week. Total hours were grouped into 6 integer values such that 1 indicated less than 1 h was spent engaged in that activity per week, 2 indicated 1 – 2½ h spent, 3 indicated 3 – 4½ h, 4 indicated 5 – 6½ h, 5 indicated 7 – 8½ h, and 6 indicated 9 or more hours (**Figure 1**). Participants are also allowed to fill in “other” and record an activity that was not listed in the questionnaire.

Physical activities were divided into light and moderate-heavy groups based on the ratio of work metabolic rate to resting metabolic rate (MET) adjusted for older adults (Ainsworth et al., 1993) as was done in the original CHAMPS research (Stewart et al., 2001). Light physical activities were those with a MET score below 30 and included conditioning training, yoga or tai chi, leisurely walking, walking to do errands, light gardening, light house work, golfing using a cart, and aerobic dancing. Moderate-heavy physical activities were those with a MET score equal to or above 30 and included sports, light or heavy strength training, swimming gently or fast, water exercises, working on aerobic machines, bicycling, fast walking, uphill walking or hiking, jogging or running, working on machinery (car, lawn mower, etc.), heavy gardening, heavy housework, singles or doubles tennis, golfing without use of a cart, dancing (such as square, folk, line, or ballroom), and skating (ice, roller, or in-line). Social activities were any for which the majority of time spent likely involved interpersonal interaction. These included visiting with family or friends, going to a senior center, volunteering, church-related activities, participating in clubs or groups, playing cards or board games with others, and shooting pool or billiards. Cognitive activities were those for which the majority of time spent was likely cognitively engaging rather than interpersonal. These included using a computer, doing arts or crafts, attending a concert, movie, lecture, or sport event, playing a musical instrument, and reading. If the “other” option was filled in, the activity described was placed in the most appropriate group. The integer measures (1 through 6) representing amount of time spent weekly in each activity were summed to create totals for light and moderate-heavy physical activities, social activities, and cognitive activities.

### Magnetic Resonance Imaging

#### Acquisition

Magnetic resonance imaging data were acquired using a Philips Achieva 3.0 Tesla scanner (Achieva; Philips Electronics, Amsterdam, The Netherlands) at the McKnight Brain Institute (University of Florida, Gainesville, FL, USA) with a standard 32-channel receive-only head coil. High-resolution 3D T1-weighted MPRAGE scans were performed. Scans were acquired in a sagittal orientation with parameters as follows: voxel size = 1 mm isotropic; 1 mm slice thickness; TE = 3.2 ms; TR = 7.0 ms; FOV = 240 × 240; Number of slices = 170.

#### Analysis

T1-weighted MRIs were automatically segmented and volumes were calculated using FreeSurfer software, version 5.3.0, available at http://surfer.nmr.mgh.harvard.edu/ (Fischl et al., 2002). Following preprocessing, all results underwent quality control to confirm correct detection of gray and white matter. Any errors in segmentation were corrected manually and the T1 images were re-processed through FreeSurfer. Seventy one percentages of cases had some form of manual edit. These consisted of adding control points to extend the white matter boundary (55%), removing voxels from the brain mask (29%), and editing the white matter mask (6%). Some subjects had edits from more than one category, such as both control points and brain mask edits. Previous research has shown that this semi-automated procedure yields accurate and reliable results when compared to manual segmentation (Fischl et al., 2002; Jovicich et al., 2009; Morey et al., 2009) and histological measures (Morey et al., 2009). Automatically parcellated FreeSurfer gray matter regions of interest (ROIs) were based on the Desikan-Killiany atlas. Intracranial volume (ICV) was calculated based on the talairach transform (Buckner et al., 2004). ROIs for the left and right hemisphere were summed and corrected for ICV (by dividing ROI volume by ICV and multiplying by 100) to create bilateral, normalized ROIs. Some of these were then summed to create volumes for the four major lobes of the brain. Specifically, the frontal ROI consisted of the caudal and rostral anterior cingulate cortices, the caudal and rostral middle frontal cortices, the lateral and medial orbitofrontal cortices, the pars orbitalis, the superior frontal cortex, and the frontal pole; the parietal ROI consisted of the precuneus, the inferior and superior parietal lobules, and the supramarginal gyrus; the temporal ROI consisted of the entorhinal cortex, the inferior, middle, and superior temporal regions, the transverse temporal region, and the temporal pole; and the occipital ROI consisted of the lateral occipital region, the lingual and fusiform gyri, the pericalcarine region, and the cuneus. All four lobes were added together to create a measure of total cortical gray matter volume. Subcortical ROIs were chosen based on their relevance to cognitive and behavioral functioning and included the thalamus, caudate, hippocampus, and amygdala.

### Statistical Analysis

All statistical analyses were performed using SPSS. Stepwise regression with backward selection was used to predict weekly time spent in either light physical, heavy physical, social, or cognitive activity using a cortical or subcortical ROI (corrected by total ICV) as well as age, education, and gender as covariates. Individual regression analyses were conducted for each ROI. In stepwise regression with backward selection, all independent variables (predictors and covariates) are entered into the equation and sequentially removed based on the probability of F. The criterion used was *p* ≥ 0.10. The first model for which all independent variables included explained significant variance in the dependent variable was chosen as the best model. The benefits of using this analytic method is that it allows for all variables to included, as it may be that a set of variables has better predictive validity than the subset, but it also removes those that may be falsely lowering the contribution of significant predictors by overlapping in variance explained. Thus, the final model efficiently explains the variance in the dependent variable and better identifies the unique contribution of the predictors.

## Results

**Table 2** displays the percentage of ICV for cortical and subcortical regions in this sample. In regards to activity measures, scores do not refer directly to number of hours, but rather to ordinal measures reflecting roughly 1.5-h increments (**Figure 1**). Though it cannot be determined exactly how much time was spent in each of the activity categories, it appears that participants divided their time roughly evenly amongst light physical (mean = 8.7, SD = 5.1), heavy physical (mean = 8.4, SD = 5.7), social (mean = 10.5, SD = 6.0), and cognitive (mean = 11.3, SD = 4.5) activities.

**Table 2:** Gray matter regions of interest presented as percentages of total intracranial volume.

Region of Interest	Mean (SD)	Range
Frontal lobe	7.83 (1.19)	6.18–11.52
Parietal lobe	5.77 (0.82)	4.34–8.35
Temporal lobe	4.75 (0.72)	3.48–6.94
Occipital lobe	4.43 (0.73)	3.43–6.33
Total cortex	22.78 (3.35)	18.14–32.43
Thalamus	0.91 (0.15)	0.71–1.35
Caudate	0.47 (0.08)	0.37–0.71
Hippocampus	0.54 (0.13)	0.30–1.01
Amygdala	0.22 (0.05)	0.13–0.37

**FIGURE 2:**
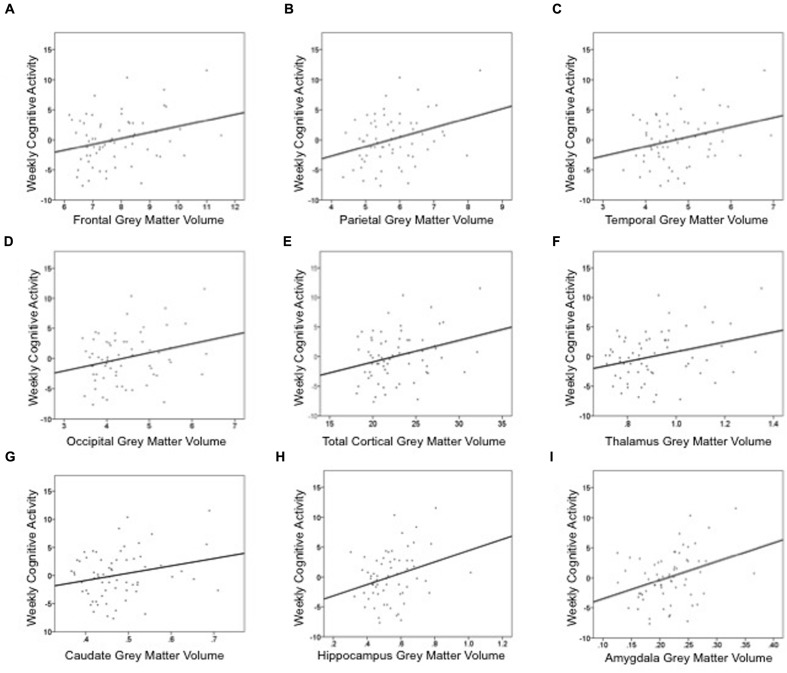
**Summed measures of weekly time spent in cognitively engaging activity are positively associated cortical and subcortical gray matter volumes: (A) frontal lobe, (B) parietal lobe, (C) temporal lobe, (D) occipital lobe, (E) total cortex, (F) thalamus, (G) caudate, (H) hippocampus, and (I) amygdala**. These graphs depict the relationship between gray matter volume and cognitive activity, controlling for education. Thus, the *y*-axis is the unstandardized residual after regressing the effects of education out of cognitive activity. Gray matter volume is a percentage of intracranial volume.

Cognitive activity was the only outcome significantly associated with gray matter volume. **Table 3** lists these final, best-fitting models. Final models revealed a positive association with education (*p* < 0.001) and all cortical and subcortical ROIs examined (*p*s < 0.05). **Figure 2** depicts the relationships between cognitive activity and brain volumes, controlling for education. In each regression, variables excluded were age and sex.

**Table 3:** Best-fitting models predicting cognitive activity from gray matter region of interest, age, sex, and education.

Model/ROI	Variable	β	*p*	*R*^2^	Model *p*	Excluded Variables
Frontal lobe	Frontal lobe	0.276	0.019ˆ*	0.247	<0.001^∗^	Age
	Education	0.493	<0.001ˆ*			Sex
Parietal lobe	Parietal lobe	0.305	0.009ˆ*	0.262	<0.001^∗^	Age
	Education	0.505	<0.001ˆ*			Sex
Temporal lobe	Temporal lobe	0.275	0.020ˆ*	0.246	<0.001^∗^	Age
	Education	0.501	<0.001ˆ*			Sex
Occipital lobe	Occipital lobe	0.256	0.030ˆ*	0.237	<0.001^∗^	Age
	Education	0.490	<0.001ˆ*			Sex
Total cortex	Total cortex	0.289	0.014ˆ*	0.253	<0.001^∗^	Age
	Education	0.502	<0.001ˆ*			Sex
Thalamus	Thalamus	0.310	0.010ˆ*	0.261	<0.001^∗^	Age
	Education	0.526	<0.001ˆ*			Sex
Caudate	Caudate	0.233	0.049ˆ*	0.227	<0.001^∗^	Age
	Education	0.482	<0.001ˆ*			Sex
Hippocampus	Hippocampus	0.286	0.017ˆ*	0.250	<0.001^∗^	Age
	Education	0.513	<0.001ˆ*			Sex
Amygdala	Amygdala	0.336	0.004ˆ*	0.280	<0.001^∗^	Age
	Education	0.519	<0.001ˆ*			Sex

*ROI, region of interest. ^∗^p < 0.05.*

In contrast, there were no significant associations observed between the volumes of these brain regions and engagement in light physical, heavy physical, or social activities as reported on the CHAMPS. More heavy physical activity was associated with younger age and male gender, whereas other activities were not associated with age or sex. Supplemental material includes these results.

## Discussion

In this study, we found that greater time dedicated to cognitively engaging activities was associated with greater cortical and subcortical brain volume. We did not find significant associations with physical or social activity level and volumetric data. Cognitive engagement activities represent one of three potential proxies for the construct of cognitive reserve. Epidemiologic-based support for the association between leisure and cognitive status has been accumulating for nearly a quarter of a century (Christensen and Mackinnon, 1993). In an effort to clarify causal attributions or directionality, other epidemiologic work has used sophisticated dynamic change models to show that *change* in cognitive leisure participation can result in higher scores on cognitive ability measures (Mitchell et al., 2012). Such evidence has sparked experimental investigations, with clinical trials demonstrating improved cognition over short periods (3–6 months) for those participating in cognitive leisure activities compared to controls (Stine-Morrow et al., 2008).

More recently, investigators have begun to examine imaging and volumetric evidence, showing that one’s level of cognitive reserve is associated with brain volume. For instance, it has been shown that a more active cognitive lifestyle is associated with greater frontal and parietal brain volume in healthy older adults (Stine-Morrow et al., 2008). Our finding that higher cognitive engagement is associated with greater hippocampal volume was also reported in a longitudinal study (Valenzuela et al., 2008). Valenzuela et al. (2008) used the Lifetime of Experiences Questionnaire (LEQ), in which sample activities included creative arts, reading, writing, and socializing. The investigators reported a significant association between total LEQ and average hippocampal volume, controlling for age, gender, hypertension, and ICV. High LEQ individuals experienced an average loss of 3.6% of hippocampal volume over a 3-year period, while low LEQ individuals exhibited more than twice this volumetric loss (8.3%). It is important to note that these studies used a composite measure to establish these associations, which, in addition to cognitive engagement activities, also included education and job complexity. Yet cognitive engagement or cognitive leisure activities show unique contributions to cognitive health, independent of the variance attributed to education and occupational status (Stern, 2009; Foubert-Samier et al., 2012). Indeed, in the current investigation, the relationship between more involvement in cognitively engaging leisure activities and greater brain volumes was not explained by education.

Findings from the current study are similar to those of another observational study in which greater frequency of cognitive leisure activities (playing games like cards, checkers, crosswords, or other puzzles) was related to better cognitive performance and reduced brain atrophy (Schultz et al., 2015). Schultz et al. (2015) reported that higher activity scores were associated with greater gray matter volumes in several ROIs including the hippocampus, posterior cingulate, anterior cingulate, and middle frontal gyrus. However, our findings differ from a longitudinal study that found no relationship between cognitive activity and MRI measures of whole brain volumes of gray and white matter (Vaughan et al., 2014). One potential reason for a lack of association in the Vaughan et al. (2014) study may be related to the items included in their 6-item measurement of cognitive activities. Like in the present study, they included reading and craft activities, but they also included group activities, social activities, and watching television. It is possible that group and social activities present with the local dependence, or relatedness; local independence, or unrelatedness, of items is a modern test theory assumption that, when violated, negatively impacts construct validity. Furthermore, television use has been shown to be negatively correlated with other cognitive activity items (Gow et al., 2012b; Fieo et al., 2014). Television time has also been associated with less frequent engagement in social and physical recreation (Hu et al., 2003) and increased risk for dementia (Rundek and Bennett, 2006). These factors may have reduced the power for detecting associations in the Vaughan et al. (2014) study. The current findings, combined with prior research in cognitive leisure activity, reinforce the relationship between hippocampal and cortical volumes and cognitive activity, consistent with our hypothesis.

It is somewhat surprising that heavy physical activity was not associated with volumetric data, as studies examining exercise effects on regional brain volume typically implicate frontal and hippocampal brain areas (Colcombe et al., 2006; Pereira et al., 2007; Erickson et al., 2011). Part of this discrepancy undoubtedly comes from the differences between intervention and observational studies. Intervention studies inherently involve introducing something novel in the lives of research participants. It may be that the amount of novelty an activity offers is more important than the type of activity itself in impacting cognitive and neurological functioning (Bielak, 2010). Thus, when intervention studies implement a physical activity regimen in sedentary adults (Colcombe et al., 2006), the novelty of the activity and significant change in lifestyle may very well impact brain structure.

Part of the discrepancy between the current findings and results from previous physical activity studies may be due to differences in methodology for measuring physical activity. The CHAMPS questionnaire asked participants to retrospectively indicate their levels of physical activity in a variety of class categories for a typical week over the previous month. Given the number of parameters that had to be remembered, it is possible that participants gave unreliable reports of their own activity (LaPorte et al., 1985). Indeed, Buchman et al. (2008) showed that objectively measured physical activity was associated with cognitive function, whereas self-reported daily physical activity in the same group was not. However, an evaluation of the CHAMPS questionnaire revealed a moderately strong correlation between self-reported engagement in moderate physical activity and activity objectively measured by a waist monitor (*r* = 0.48) (Harada et al., 2001). Furthermore, other self-report studies have shown associations between physical activity levels and cortical volumes (Erickson et al., 2010; Bugg and Head, 2011; Gow et al., 2012a; Benedict et al., 2013). As such, it is unlikely that the lack of association is entirely explained by the self-report nature of the physical activity measurement.

Our findings are consistent with Gow et al. (2012a), who measured baseline physical activity via self-report at age 70 years and administered MRIs at age 73. They found physical activity to be associated with white matter volume, but not gray matter volume. Thus, lack of findings in the present study may reflect the fact that only gray matter volume was measured. Given that the current findings are inconsistent with a large body of research on the impact of physical activity on brain volumes, we do not feel comfortable rejecting our hypothesis that greater physical activity is associated with greater volumes, particularly in frontal and limbic regions.

Social activity is more tenuously linked to brain volume than are physical and cognitive activity, and the relationship was not supported in the current investigation. Greater social involvement has been associated with greater normal-appearing white matter (Gow et al., 2012a), total brain (Mortimer et al., 2012), and gray matter (James et al., 2012) volumes. Yet methodological differences may explain discrepancies between prior results and our own.

Gow et al. (2012a) combined cognitive and social leisure activities when measuring the link between activity and brain structure. While greater cognitive and social activity was related to more normal-appearing white matter, these activities were not found to be associated with gray matter volume, consistent with the present data. Whether social activity has a stronger relationship to white matter than to gray matter volume deserves further investigation.

Mortimer et al. (2012) performed an intervention study and found that their social interaction groups showed significant increases in total brain volume over the study period. The fact that this study was an intervention may explain why their results differ from the current study, since, as previously discussed, the amount of novelty provided by an intervention may be a significant driver of results (Bielak, 2010). The intervention group met three times per week at a neighborhood community center, the participants decided on their own to organize and select topics of conversation, and the discussions were described as extremely lively. Thus, lifestyle was significantly impacted in these participants.

James et al. (2012) demonstrated that higher social engagement was associated with greater total brain volume and total gray matter volume, as well as greater temporal and occipital gray matter lobar volumes. Yet methodological differences might also explain the discrepancies between their results and our own. The James et al. study included a question more related to physical than social activity in their eight-item measure of social engagement, as they asked participants how many times in the past week/month they had done any indoor or outdoor recreational activity like bowling, working out, fishing, hiking, boating, swimming, or golfing. Given that physical activity has a more robust association to gray matter volume than does social activity, responses to this question may have been a strong driver of results. Another important difference is in the study samples; the James sample was, for the most part, comprised of former lead workers who were recruited for a study of lead exposure and cognitive function. They were less socially engaged than population-based controls, who comprised 12% of the sample. Not only were the participants in the current study recruited from the same community population, but the sample size was significantly smaller compared to the James et al. sample of 348. Thus, differences in population and sample size may have given James et al. more statistical power to detect any relationship between the measured activities and gray matter volume.

Our null findings for social activity are similar to those of Foubert-Samier et al. (2012), who assessed activity in midlife and in retirement. They asked participants whether or not they engaged in a number of activities during midlife (when they worked) and currently (in retirement) and assigned one point for each activity. Social activity in both midlife and retirement was related to better semantic verbal fluency, but unrelated to total brain, gray matter, or white matter volumes.

Current findings are also consistent with Van der Vegt (2015), who found no association between social activity with family and friends and brain volume (Van der Vegt, 2015). In that study, participants were asked two questions: “In general, how often do you have contact with your family members (including telephone calls or letters)?” and, “In general, how often do you have contact with your friends or well-known acquaintances (including telephone calls or letters)?” Interestingly, factor analytic methods have shown that social activity can be presented as a bi-factor model: social-private and social-public (Jopp and Hertzog, 2010). Additionally, social-private was more highly correlated with cognitive functioning measures. As such, social-private may have a stronger relationship with brain volume than social private, and combining separable constructs may negatively impact predictive validity. The association between different types of social activity and volumetric data of cortical and subcortical regions warrants further study. Nevertheless, considering prior research and the results of the current study, we cannot support our hypothesis that social activity is associated with greater gray matter volumes in temporal and occipital regions.

As mentioned previously, novelty may be an important driving factor in the relationship between leisure activities that are engaging, cognitively or otherwise, and larger brain volumes. Animal models have shown that, in aged animals, environmental enrichment attenuates the age-related changes in cortical thickness (Mora et al., 2007). A defining feature of enrichment in animals is often novelty. For instance, repeatedly substituting and replacing the objects in the home cages creates a wide range of opportunities for enhanced cognitive stimulation, formation of tuned spatial maps, and proficient detection of novelty (Petrosini et al., 2009). In humans, one theoretical explanation for how novelty exerts a protective effect in older adults relates to non-adaptive “routinization” (Tournier et al., 2012). That is, as older adults continue to accumulate age-related insults (e.g., medical comorbidities or muscle related fatigue), some individuals may seek out more controlled, stable, predictive environments, thus limiting exposure to novel environments or experiences. Evidence in support of this can be found in a Bergua et al. (2006) study, demonstrating that preferences for routine were positively correlated with cognitive decline over 3 years. Additional support can be observed in the finding that greater variety of participation in cognitively stimulating activities was associated a ∼10% lower risk of cognitive impairment, regardless of how challenging these tasks were (Carlson et al., 2012). As such, though novel environments and situations may be more difficult to navigate as people age, they may be the most protective against neurological decline.

### Limitations

The most common grouping scheme for investigating the efficacy of leisure activities has been the broad domains of physical, social activities, and other cognitive leisure activities, which is what we followed for this investigation. However, with such broad domains, many activities cannot be assigned unambiguously. Volunteering work, for instance, is likely to have a strong social component, but may in some instances entail more non-verbal cognitive or physical effort. Ideally, large item/activity pools would allow for the formation of more distinct, unidimensional activity constructs, for example, further delineating social activities into social-private and social-public (Jopp and Hertzog, 2010). Furthermore, historically, the evidence in observational studies, including the present study, has been limited to an examination of frequency of participation. However, contemporary conversations have moved beyond questioning the frequency of participation, placing greater emphasis on novelty/variety and cognitive challenge (Bielak, 2010; Chan et al., 2014). Finally, as previously mentioned, the accuracy of measurement in self-report questionnaires is questionable, as participants may have subjective biases and may, intentionally or unintentionally, misrepresent their objective activity levels (Erickson et al., 2012). Nevertheless, CHAMPS has several strengths, including robust psychometric properties (Harada et al., 2001; Wilcox et al., 2009), sensitivity to change (Stewart et al., 1997), and correlations with objective measures of physical activity and functioning (King et al., 2000; Harada et al., 2001; Stewart et al., 2001).

## Conclusion

Results of this study emphasize the relationship between volumetric data and the independent contribution of cognitively engaging activities. This process is of considerable value if we consider that cognitive leisure activities show unique contributions to cognitive health, independent of the variance attributed to other cognitive reserve proxies (e.g., education and occupational status) (Stern, 2009; Foubert-Samier et al., 2012). Results of the present study show that more time spent engaged in cognitive activity is associated with greater brain volume. Importantly, these lifestyle variables are modifiable, suggesting that interventions aimed at increasing levels of cognitive activity may delay onset or decrease risk of developing dementia. In fact, intervention studies have shown positive neurological effects of cognitive interventions. Still, the field of cognitive aging will benefit from more research investigating interventions aimed at increasing everyday cognitively engaging activities as well as the role of other biomarkers such as inflammatory agents and genetic factors in influencing brain structure and function.

## Author Contributions

TS was involved in the conception and writing of the manuscript and conducted statistical analyses. RF was involved in the writing of the manuscript. AO conducted volumetric analyses that were used in the manuscript. EP, AW, and RC were involved in the conception of the study, and RC was involved in the conception of the manuscript and data analysis. All authors contributed intellectual content and approved the version to be published.

## Conflict of Interest Statement

The authors declare that the research was conducted in the absence of any commercial or financial relationships that could be construed as a potential conflict of interest.
