# Supplementary material for: Cognitively Engaging Activity Is Associated with Greater Cortical and Subcortical Volumes
Source: Front Aging Neurosci. 2016 May 2;8:94. doi: 10.3389/fnagi.2016.00094 (PMC4852201; doi:10.3389/fnagi.2016.00094)
Supplement: Supplementary file 1 [file Table_1.DOCX]

Supplementary Table 1: Initial models predicting light physical activity from grey matter region of interest, age, sex, and education. Final models not reported because all variables were excluded.

| Model/ROI | Variable | *β* | *p* | *R*^2^ | Model *p* |
| --- | --- | --- | --- | --- | --- |
| Frontal lobe | Frontal lobe | .071 | .644 | .081 | .273 |
|  | Age | .232 | .117 |  |  |
|  | Sex | .203 | .137 |  |  |
|  | Education | -.029 | .828 |  |  |
| Parietal lobe | Parietal lobe | -.091 | .569 | .083 | .262 |
|  | Age | .151 | .328 |  |  |
|  | Sex | .240 | .077 |  |  |
|  | Education | -.043 | .749 |  |  |
| Temporal lobe | Temporal lobe | .079 | .613 | .082 | .269 |
|  | Age | .237 | .113 |  |  |
|  | Sex | .202 | .139 |  |  |
|  | Education | -.026 | .845 |  |  |
| Occipital lobe | Occipital lobe | -.035 | .817 | .078 | .290 |
|  | Age | .184 | .212 |  |  |
|  | Sex | .228 | .092 |  |  |
|  | Education | -.038 | .774 |  |  |
| Total cortex | Total cortex | .014 | .929 | .078 | .294 |
|  | Age | .207 | .173 |  |  |
|  | Sex | .217 | .112 |  |  |
|  | Education | -.033 | .802 |  |  |
| Thalamus | Thalamus | -.072 | .639 | .081 | .272 |
|  | Age | .170 | .241 |  |  |
|  | Sex | .235 | .082 |  |  |
|  | Education | -.048 | .724 |  |  |
| Caudate | Caudate | .153 | .258 | .097 | .182 |
|  | Age | -.011 | .074 |  |  |
|  | Sex | .197 | .134 |  |  |
|  | Education | .240 | .935 |  |  |
| Hippocampus | Hippocampus | .100 | .536 | .083 | .256 |
|  | Age | .249 | .103 |  |  |
|  | Sex | .196 | .151 |  |  |
|  | Education | -.022 | .872 |  |  |
| Amygdala | Amygdala | .113 | .488 | .085 | .247 |
|  | Age | .260 | .097 |  |  |
|  | Sex | .194 | .153 |  |  |
|  | Education | -.024 | .857 |  |  |

ROI = Region of interest
