# Supplementary material for: Cognitively Engaging Activity Is Associated with Greater Cortical and Subcortical Volumes
Source: Front Aging Neurosci. 2016 May 2;8:94. doi: 10.3389/fnagi.2016.00094 (PMC4852201; doi:10.3389/fnagi.2016.00094)
Supplement: Supplementary file 2 [file Table_2.DOCX]

Supplementary Table 2: Best-fitting models predicting heavy physical activity from grey matter region of interest, age, sex, and education.

| Model/ROI | Variable | *β* | *p* | *R*^2^ | Model *p* | Excluded Variables |
| --- | --- | --- | --- | --- | --- | --- |
| Frontal lobe | Age | -.234 | .049* | .194 | .001* | Frontal lobe |
|  | Sex | -.411 | .001* |  |  | Education |
| Parietal lobe | Age | -.234 | .049* | .194 | .001* | Parietal lobe |
|  | Sex | -.411 | .001* |  |  | Education |
| Temporal lobe | Age | -.234 | .049* | .194 | .001* | Temporal lobe |
|  | Sex | -.411 | .001* |  |  | Education |
| Occipital lobe | Age | -.234 | .049* | .194 | .001* | Occipital lobe |
|  | Sex | -.411 | .001* |  |  | Education |
| Total cortex | Age | -.234 | .049* | .194 | .001* | Total cortex |
|  | Sex | -.411 | .001* |  |  | Education |
| Thalamus | Age | -.234 | .049* | .194 | .001* | Thalamus |
|  | Sex | -.411 | .001* |  |  | Education |
| Caudate | Age | -.234 | .049* | .194 | .001* | Caudate |
|  | Sex | -.411 | .001* |  |  | Education |
| Hippocampus | Age | -.234 | .049* | .194 | .001* | Hippocampus |
|  | Sex | -.411 | .001* |  |  | Education |
| Amygdala | Age | -.234 | .049* | .194 | .001* | Amygdala |
|  | Sex | -.411 | .001* |  |  | Education |

ROI = Region of interest

Sex coded as Male = 1, Female = 2

* *p* < .05
