# Supplementary material for: Cognitively Engaging Activity Is Associated with Greater Cortical and Subcortical Volumes
Source: Front Aging Neurosci. 2016 May 2;8:94. doi: 10.3389/fnagi.2016.00094 (PMC4852201; doi:10.3389/fnagi.2016.00094)
Supplement: Supplementary file 3 [file Table_3.DOCX]

Supplementary Table 3: Initial models predicting social activity from grey matter region of interest, age, sex, and education. Final models not reported because all variables were excluded.

| Model/ROI | Variable | *β* | *p* | *R*^2^ | Model *p* |
| --- | --- | --- | --- | --- | --- |
| Frontal lobe | Frontal lobe | .072 | .650 | .014 | .929 |
|  | Age | .076 | .614 |  |  |
|  | Sex | .083 | .553 |  |  |
|  | Education | .009 | .945 |  |  |
| Parietal lobe | Parietal lobe | .077 | .644 | .014 | .928 |
|  | Age | .085 | .596 |  |  |
|  | Sex | .085 | .541 |  |  |
|  | Education | .010 | .941 |  |  |
| Temporal lobe | Temporal lobe | .045 | .779 | .012 | .946 |
|  | Age | .065 | .671 |  |  |
|  | Sex | .090 | .520 |  |  |
|  | Education | .009 | .951 |  |  |
| Occipital lobe | Occipital lobe | .003 | .986 | .011 | .956 |
|  | Age | .045 | .765 |  |  |
|  | Sex | .101 | .468 |  |  |
|  | Education | .004 | .978 |  |  |
| Total cortex | Total cortex | .056 | .733 | .013 | .941 |
|  | Age | .071 | .647 |  |  |
|  | Sex | .088 | .530 |  |  |
|  | Education | .009 | .949 |  |  |
| Thalamus | Thalamus | -.011 | .943 | .011 | .956 |
|  | Age | .039 | .792 |  |  |
|  | Sex | .103 | .455 |  |  |
|  | Education | .001 | .992 |  |  |
| Caudate | Caudate | .126 | .368 | .024 | .828 |
|  | Age | .077 | .577 |  |  |
|  | Sex | .081 | .548 |  |  |
|  | Education | .023 | .867 |  |  |
| Hippocampus | Hippocampus | .243 | .143 | .046 | .581 |
|  | Age | .163 | .291 |  |  |
|  | Sex | .041 | .764 |  |  |
|  | Education | .036 | .794 |  |  |
| Amygdala | Amygdala | .207 | .218 | .036 | .696 |
|  | Age | .154 | .335 |  |  |
|  | Sex | .053 | .704 |  |  |
|  | Education | .023 | .864 |  |  |

ROI = Region of interest
